# Supplementary material for: Medium-Term Clinical Outcomes of the Global Icon Stemless Shoulder System: Results of a 2-Year Follow-Up
Source: J Clin Med. 2023 Oct 25;12(21):6745. doi: 10.3390/jcm12216745 (PMC10648431; doi:10.3390/jcm12216745)
Supplement: Supplementary file 1 [file jcm-12-06745-s001.zip › jcm-2648131-supplementary.pdf]

Supplementary File

Table S1. Sub-Sections of the WOOS: Within- Subject Contrasts

	**Mean (SD)**	**Std. Error**	**95 % CI**	**F Score**	**Effect Size (Partial eta)**	**P value**
WOOS Index Physical						
Baseline	347.4 (117.9)	35.5	268.2 to 426.6			
12 months	77.6 (91.2)	27.5	16.3 to 138.9			
24 months	72.6 (111.7)	33.7	-2.4 to 147.7	85.1	0.895	**<0.001**
WOOS Index Recreation/Sport						
Baseline	353.7 (91.9)	27.7	292 to 415.4			
12 months	95.7 (124)	37.4	12.4 to 179			
24 months	78.2 (96.8)	29.2	13.1 to 143.2	82.0	0.891	**<0.001**
WOOS Index Lifestyle						
Baseline	368.0 (101.6)	30.6	299.7 to 436.3			

12 months	97.7 (118.5)	35.7	18.1 to 177.3			
24 months	73.1 (120.8)	36.4	-8.0 to 154.2	70.8	0.876	**<0.001**
WOOS Index Emotion						
Baseline	198.9 (59.5)	17.9	158.9 to 238.9			
12 months	48.1 (74.6)	22.5	-2.0 to 98.2			
24 months	44.2 (74.2)	22.4	-5.6 to 94.0	67.2	0.871	**<0.001**

Note. WOOS Index = Western Ontario Osteoarthritis Shoulder Index. Significance set at 0.05. p values in bold indicate statistical significance.

Table S2. Sub-Sections of the WOOS Index: Effects between time points.

	Time Point Comparisons		Mean Difference (Std. Error)	Effect Size (95 % CI)	t value	P value
WOOS Index physical	Baseline	12 months	269.7 (41.6)	106. 8 (177.0 to 362.5)	13.7	**<0.001**
		24 months	274.7 (29.8)	90.6 (208.4 to 341.1)	11.0	**<0.001**
	12 months	24 months	5.0 (39.7)	131.6(-83.4 to 93.4)	0.1	0.90
WOOS Index Rec/Sport	Baseline	12 months	258.0 (38.4)	104.4 (172.5 to 343.5)	13.2	**<0.001**
		24 months	275.5 (30.4)	96.4 (207.8 to 343.3)	9.9	
	12 months	24 months	17.5 (35.8)	118.8 (-62.3 to 97.4)	0.5	0.64
WOOS Index Lifestyle	Baseline	12 months	270.3 (31.7)	93.1 (119.7 to 340.8)	15.7	**<0.001**
		24 months	294.9 (35.1)	107/2 (216.8 to 373.0)	9.7	**<0.001**
	12 months	24 months	24.6 (32.1)	106.5 (-46.9 to 96.2)	0.8	0.46
WOOS Index Emotion						
	Baseline	12 months	150.8 (20.9)	67.3 (104.4 to 197.3)	10.7	**<0.001**
		24 months	154.7 (18.9)	60.5 (112.7 to 196.8)	8.7	**<0.001**
	12 months	24 months	3.9 (13.3)	44.2 (-25.8 to 33.6)	0.3	0.78

Note. WOOS= Western Ontario Osteoarthritis Shoulder Index. Significance set at 0.05. p values in bold indicate statistical significance.
